# Inoculation of the fungi-static *C. iranensis* ZJW-6 accelerates compost maturation, enhances fertilizer efficiency, and increases the abundance of target microbes in spring and autumn compost

**DOI:** 10.3389/fmicb.2025.1592864

**Published:** 2025-05-22

**Authors:** Dongchao Wang, Jingqi He, Liangdong Li, Gaoyuan Wu, Zixian Jiang, Baifeng Cang, Meikang Wu, Min Nuo, Zichen Liu, Jiaxuan Li, Shengbo Xu, Xintong Ma, Zhihai Wu, Meiying Yang

**Affiliations:** ^1^Faculty of Agronomy, Jilin Agricultural University, Changchun, China; ^2^College of Life Sciences, Jilin Agricultural University, Changchun, China

**Keywords:** composting, lignocellulose degradation, humic acid, bacterial community, Actinobacteriota

## Abstract

**Introduction:**

Straw composting is an effective way of straw resource utilization, and inoculation of microorganisms can accelerate straw decomposition. This study initially investigated the effects of adding fungi-static *C. iranensis* (ZJW-6) on lignocellulose, humic acid, and bacterial communities during the spring and autumn composting of rice straw in Northeast China.

**Methods:**

ZJW-6 is a cellulose degrading bacterium, which is used as the microorganism for composting inoculation. Four treatments are set up:spring composting with no bacteria added; spring composting with added bacteria, autumn composting without added bacteria, and autumn composting with added bacteria. The effects of adding microorganisms in spring and autumn composting on its physical and chemical properties and microbial community changes are explored.

**Results:**

The results demonstrated that ZWJ-6 altered the bacterial community composition by increasing the relative abundance of lignocellulose-decomposing bacteria and the Actinobacteriota phylum in both spring and autumn composting. This enhancement strengthened the functional contributions of the bacterial community during composting. Specifically, the degradation efficiencies of lignin, cellulose, and hemicellulose in straw composting increased by 7.63%–14.71%, 22.45%–97.76%, and 28.48%–41.71%, respectively. Additionally, the content of humic acid and nitrogen increased by 12.44%–38.27% and 4.56%–5.81%, respectively. Autumn composting showed better decomposition and maturity.

**Discussion:**

The addition of ZJW-6 promotes the maturation of both spring and autumn compost decomposition, offering a new agricultural solid waste disposal options for the straw-returning cultivation model in the region. Overall, adding ZJW-6 to autumn straw compost is more suitable for rice-growing areas in northeastern China.

## 1 Introduction

The world produces approximately 3.6 billion tons of crop residue annually. China alone accounts for a quarter of this amount (Mengmeng et al., 2022). Improper disposal of this residue can lead to significant ecosystem hazards (Xinya et al., 2024). Straw is a valuable organic resource (Li et al., 2020), rich in nitrogen, phosphorus, potassium, organic matter, and trace elements. It can be recycled through composting, converting the straw into organic fertilizer (Shunyao et al., 2022). However, crop straw contains high levels of organic carbon components (lignin, cellulose, and hemicellulose), which are difficult to decompose. These components can only be degraded by microorganisms. These microorganisms convert them into humus and other nutrients (Wu et al., 2017). As a result, the degradation efficiency of the organic carbon components is low (Ning et al., 2021b). This significantly limits the composting decomposition process.

Cumulative temperatures vary significantly across China’s geographic regions. As a result, crop growth cycles and farming practices differ from one area to another. For example, the rice and corn in the northeast mature annually, while crops in the North China Plain mature biannually. Additionally, the practice of returning straw to the field differs between autumn and spring (Liu et al., 2024; Lou et al., 2024). These differences in climatic conditions and farming habits result in variations in the time required for straw degradation, and in cold regions, microbial activity and metabolic processes are affected by low temperatures, thus slowing down compost fermentation (Du et al., 2025). To speed up this process and accommodate different straw-return methods, exogenous substances are often added. While most studies focus on straw degradation through composting methods, there is disagreement regarding the optimal timing and duration for composting (Ning et al., 2021a; Liangcai et al., 2022; Yao et al., 2024). Therefore, research on spring and autumn composting is crucial for determining the appropriate timing for determining the best timing for returning straw to fields in Northeast China.

Given the two aforementioned issues, there is an urgent need for new technologies, materials, and methods to accelerate straw decomposition and enhance the efficiency of straw degradation. Most current composting studies focus on accelerating straw degradation by inoculating exogenous microorganisms and enhancing their activity to produce a variety of enzymes (Ricardo et al., 2022; Zhuangzhuang et al., 2023; Wang et al., 2024). These include bacterial microorganisms such as Cellulomonas (Lei et al., 2023) and Bacillus (Ontañon et al., 2021), as well as fungal microorganisms like Anaerobic (Tamilselvan and Immanuel, 2024) and Basidiomycotina (Xi et al., 2015). Among these, cellulose-degrading bacteria are particularly valuable for converting crop waste straw (Kavitch et al., 2022). Examples, such as Cellulomonas ATCC 484 (Wakarchuk et al., 2017) and *C. fimi* B-402 (Ontañon et al., 2021) are highly efficient cellulolytic agents with strong commercial potential. Their cellulolytic capacity mainly stems from microorganisms that produce lignocellulolytic enzymes or their derivatives, such as xylanase, *β*-glucosidase, etc. These enzymes degrade most of the straw’s organic carbon into humus (HS) and improve compost maturity (Wu et al., 2017; Rastogi et al., 2020). However, studies investigating the effects of inoculating cellulose-degrading bacteria on degradation outcomes and microbial communities over different composting durations remain limited.

In summary, this study uses the cellulose-degrading bacterial agent fungi-static *C. iranensis* (ZJW-6) as the inoculant. It also uses rice straw as the composting material in both spring and autumn, taking into account regional differences and varying cultivation return practices. This was the first study comparing ZJW-6 inoculation in both spring and autumn composting. ZJW-6 showed lignocellulose degradation ability under both anaerobic and aerobic conditions, and the straw degradation rate reached 54.8%. This study aims to achieve the following objectives: (1) Examine the differences in physicochemical properties between spring and autumn composting; (2) Analyze the effects of changes in maturity indicators between spring and autumn composting; (3) Explore the relationship between the metabolic characteristics of microbial communities. In addition, it aims to shorten the straw degradation time and improve compost maturation quality by adding the fungi-static *C. iranensis*, thereby addressing the regional agricultural production needs for straw return in Northeast China.

## 2 Materials and methods

### 2.1 Composting process and sampling

The fungi-static *C. iranensis* (ZJW-6) was provided by the Microbiology Laboratory, College of Life Sciences, Jilin Agricultural University. while the rice straw was obtained from the university’s experimental field.

The composting trial was conducted from October 2023 to May 2024 at Jilin Agricultural University, Changchun, China. Two composting periods were set up: spring composting (6 April 2024) and autumn composting (28 October 2023). Two sets of treatments were also applied: 1.0% bacteria (volume/wet weight of compost samples) added (BC) and no bacteria added (CK), This resulted in a total of four treatments, namely spring composting with no bacteria added (SCK), spring composting with added bacteria (SBC), autumn composting without added bacteria (ACK), and autumn composting with added bacteria (ABC). The concentration of the liquid fungicide was 1.0 × 10^9^ CFU mL^−1^ (Pre-experimental tests). Finally, the moisture content was adjusted to approximately 60%, and the C/N ratio in the compost was adjusted to 30 by adding urea. Compost sizing was performed as described by Koki (2020) with some modifications. The compost was formed into a cone shape with a height of 1.50 m and a diameter of 3.00 m at the base. A plastic film was placed at the bottom to separate the compost from the soil, which was covered after the pile was made to allow for natural fermentation.

The compost was manually turned to provide aeration when its temperature exceeded 50°C. The mixture was turned twice a week for the first 2 weeks and once a week thereafter. The center temperature of the compost was monitored daily using an automatic thermometer. Previous experiments have shown that prolonging the heating and thermophilic phases of composting enhances the efficiency of lignocellulosic biomass composting. Based on these findings, the composting process in this study was divided into five phases according to temperature changes: the initiation phase (IP), the heating phase (HP, compost temperature up to 50°C), the thermophilic phase (TP, >50°C), the cooling phase (CP, 40°C–50°C), and the maturation phase (MP, 20°C–40°C). Autumn composting has 2 additional phases (HP and CP) compared to spring composting since it is carried out at the end of October and continues throughout the winter period. Nine random samples (three from each section) were collected separately from the upper, middle, and lower layers of each pile. Approximately 500 g of samples were collected from each compost heap at each sampling time (Yao et al., 2024). The fresh samples were gently mixed and divided into two parts. One part was stored at −80°C for subsequent molecular biology analyses and enzyme activity measurements. The other part was naturally dried in the shade and used to determine physicochemical properties.

### 2.2 Physicochemical and humification analysis

The compost temperature was measured and recorded twice a day (at 10:00 and 15:00) using a stainless steel compost thermometer (KT, BJ8A, Shanghai, China) about 1.50 meters in length. Compost samples were taken from the upper (0–50 cm), middle (50–100 cm), and lower (100–150 cm) layers. The average temperature for each layer was calculated by averaging the two temperature measurements taken at those points. Total carbon (TC) and total nitrogen (TN) contents were measured using an automatic elemental analyzer (Vario EL III, Elementar, Germany), and the outputs were used to calculate the C/N ratio. pH and electrical conductivity (EC) were measured with a pH meter (PB10, Sartorius, Germany) and a conductivity meter (LF91, Werkstatten, Germany). Ammonium and nitrate nitrogen contents were determined using an autoanalyzer (Auto Analyzer3, Bran and Luebbe, Germany).

Humic substances were classified into two main fractions—humic acid and fulvic acid—based on molecular weight and functional carbon content. Fresh samples (2 g each) were used for humus extraction. Humic acid and fulvic acid were then extracted and separated from the compost heap. The organic carbon content of the humus components was measured with an organic carbon analyzer (MultiN/C-3100, Germany) as an indicator to characterize their concentration.

The lignin, cellulose, and hemicellulose contents were determined using the Lignin (model:BYSH-0708 W, 96 t), Cellulose (model:BYSH-0715 W, 96 t), and Hemicellulose (model:BYSH-0716 W, 96 t) content Determination Kit (Byabscience Biotechnology Co., Ltd.). The lignin kit uses an acetylation method to acetylate the phenolic hydroxyl groups in lignin, and finally colorimetric detection. Cellulose is hydrolyzed to glucose by heating under acidic conditions. Furfural compounds are then formed in the presence of concentrated sulfuric acid. An anthrone reagent is utilized to react with the furfural compound to produce a blue-green substance, finally colorimetric detection. Hemicellulose is hydrolyzed by heating under acidic conditions to hydrolyze hemicellulose, and finally colorimetric detection.

### 2.3 Enzyme activity measurement

Laccase, xylanase, and *β*-glucosidase activities were determined using the Laccase (model:MK10542A, 96 t), xylanase (model:MK30035A, 96 t), and β-glucosidase (model:MK30025A, 96 t), Activity Assay Kit (Jiangsu Su Enzyme Tech Biotechnology Co., Ltd.). The kit uses a double-antibody one-step sandwich enzyme-linked immunosorbent assay (ELISA). To the coated microtiter wells pre-coated with antibodies against Laccase, xylanase and β-glucosidase, respectively, the specimen, standard and HRP-labeled detection antibody were added sequentially, warmed and washed thoroughly. The color was developed with the substrate TMB, which was converted to blue by catalysis of peroxidase and to final yellow by acid, and finally colorimetric detection.

### 2.4 Microbial community structures analysis in compost

The collected samples were placed into sterile tubes, quickly stored in liquid nitrogen, and ground into a powder using a mortar and pestle. The powder was then sieved through a 20-mesh sieve to remove other impurities and transferred into sterile sampling bags. DNA extraction was performed using the Fast DNA® SPIN Kit (MP Solon, United States). The extracted DNA was detected and analyzed using gel electrophoresis. The primers 515F (5′-GTGCCAGCMGCCGCGG-3′) and 907R (5′-CCGTCAATTCMTTTRAGTTT-3′) targeting the bacterial 16 s rRNA V4-V5 region were selected for amplification. The amplification results were subjected to 2% agarose gel electrophoresis, and the target fragments were cut and purified suing the GeneJET Gel Recovery Kit (Thermo Scientific). The library was constructed using the NEB Next® Ultra™ DNA Library Prep Kit for Illumina (New England Biolabs). After quantification with Qubit and library quality assessment, the library was sequenced using the HiSeq platform.

### 2.5 Statistical analysis

The experiment was repeated three times for each set of physicochemical data and the data were expressed as average values. Physicochemical parameters were analyzed with Origin 2024 and all statistical analyses (t-test) were performed with SPSS 22.0. Significant differences in this study were set at *p* < 0.05. The R (version 0.9.2) was used to evaluate the relationships between microbial communities and the environment, perform redundancy analysis (RDA), and generate heatmaps. On the core bacterial community network analysis was performed with Gephi (version 0.9.3).

## 3 Results and discussion

### 3.1 Variation in physicochemical properties during composting

**Figure 1:**
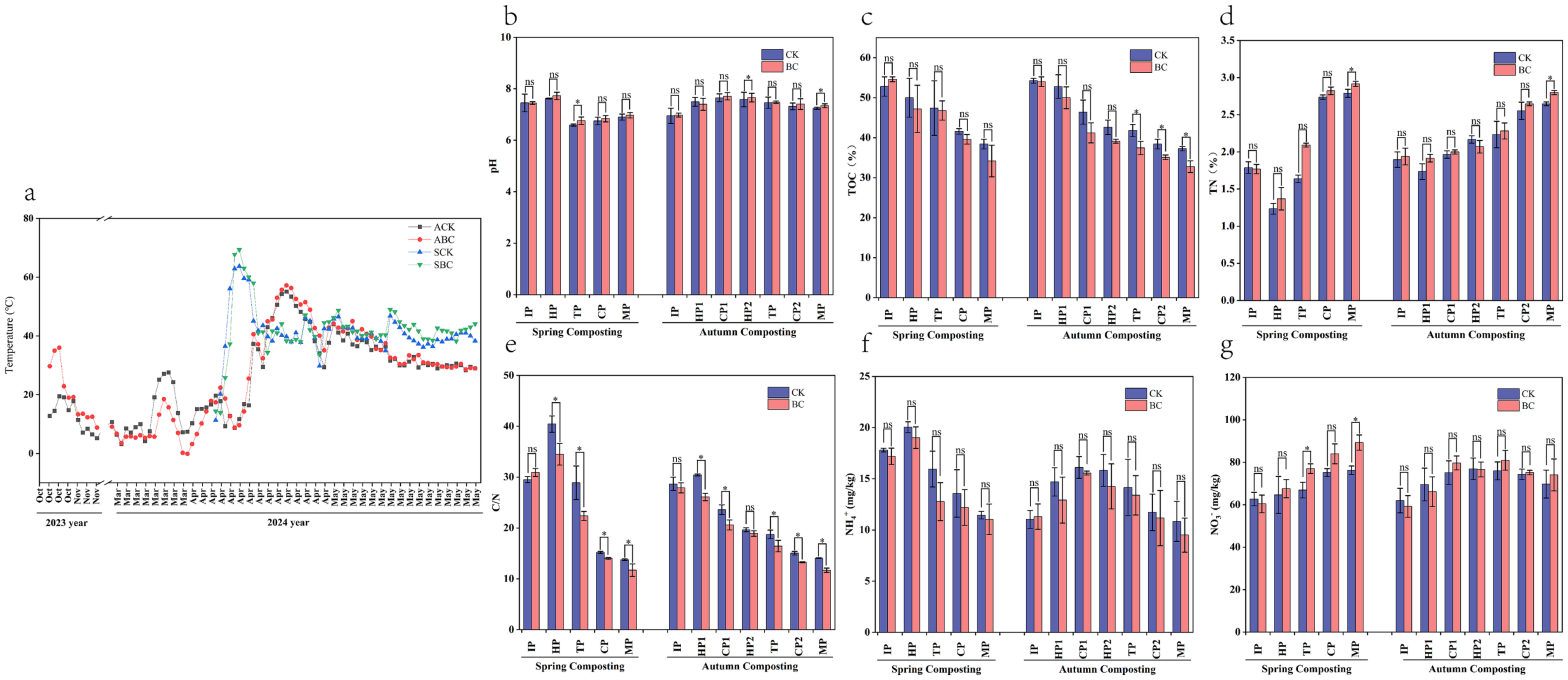
Variation of physicochemical indicators in spring and autumn compost. **(a)** Temperature, **(b)** pH, **(c)** total organic carbon, **(d)** total nitrogen, **(e)** C/N, **(f)** ammonium nitrogen, **(g)** nitrate nitrogen. ^*^Represent significant differences at *p* < 0.05 level.

Temperature is a key indicator for evaluating the compost fermentation process (Wei et al., 2018). The autumn composting and spring composting trials lasted 90 and 56 days, respectively. In the autumn composting experiment, the temperature began to rise in the initial phase in October 2023. It reached its peak on day 3. The maximum temperature in the ABC treatment was 6.5°C higher than in the ACK treatment. This difference was due to microbial activity breaking down the straw and generating significant heat (Krishnan et al., 2017). Subsequently, the temperature gradually declined as the external ambient temperature dropped to around 0°C. It remained low until it began to rise again in March of the following year, the temperature peak occurred at 50 days of the experiment, and then the temperature started to decrease and gradually stabilized (Figure 1a). In the spring composting experiment, the highest temperature was recorded on day 6. In contrast, the autumn composting experiment took 10 extra days to reach its peak temperature, and the temperature is 8.6°C–12.2°C lower. Both treatments maintained temperatures between 50°C and 60°C for 5–7 days, with the SBC treatment being 0.9°C–5.7°C higher than the SCK treatment. This indicates that the addition of ZJW-6 effectively increased the composting temperature. The last temperature peak occurred on day 38 of the spring composting experiment. The temperature then gradually dropped and eventually stabilized as the composting process was completed.

The pH trends were similar for both the spring and autumn composting treatments (Figure 1b). During the early composting stages, pH increased due to the production of ammonia gas and water-soluble ammonia from microbial decomposition of lignocellulose (López-Cano et al., 2016), and the increase in ammonium nitrogen (Figure 1f) supports the ammonia production. As decomposition progressed, ammonification decreased and lignocellulose was broken down into phenolic acid compounds (Ning et al., 2021a), causing the ammonium nitrogen and pH to decrease. In the later stages of composting, compost treated with bacteria consistently showed higher pH levels compared to compost without bacteria treatment. This aligns with the findings of previous studies (Liangcai et al., 2022). These studies suggest that the addition of exogenous microorganisms enhances the production of other derived substances in the compost.

Microorganisms require energy for their activities. Certain organic components involved in the compost degradation process provide essential nutrients for them (Solaiman et al., 2019). This leads to a continuous decline in organic carbon during composting (Figure 1c). The accelerated straw degradation during the initial warming phase of composting is driven by microbial metabolic activity. By the end of the composting process, the total organic carbon in the SBC and ABC treatments decreased by 37.44% and 39.33%, respectively. These reductions were significantly higher than those observed in SCK and ACK treatments, which were 27.27% and 31.32%, respectively. This indicates that the addition of ZJW-6 enhances microbial metabolic activity during composting.

The total nitrogen content of both spring and autumn compost increased upon the completion of composting. Compared to SCK and ACK, the nitrogen content of SBC and ABC was significantly (*p* < 0.05) higher by 4.56% and 5.81%, respectively (Figure 1d). This increase was due to the mineralization of compost or the degradation of organic matter (Zhang et al., 2014). These findings suggest that the addition of ZJW-6 promotes the accumulation of nitrogen in the compost. The total N content in spring compost sharply decreased during the HP stage, likely due to the release of NH_3_ following the initial warming. Compared to ACK and ABC, SCK and SBC exhibited 5.40% and 4.15% higher nitrogen content, which is more favorable for nitrogen retention after composting is complete.

The C/N ratio is a key indicator used to evaluate the degree and quality of compost decomposition (Onwosi et al., 2017). It plays a crucial role in ensuring the success of composting (Mohd et al., 2022). Overall, the compost C/N ratio exhibited a decreasing trend (Figure 1e), This trend was attributed to microbial activity consuming carbon sources (such as sugars, amino acids, etc.), degradation of organic matter, and CO2 emissions (Yang et al., 2019). The final C/N ratios for SCK, ACK, SBC, and ABC were 13.77, 14.08, 11.71, and 11.70, respectively. The C/N ratios of spring and autumn compost with bacteria were significantly (*p* < 0.05) lower than those without bacteria, with reductions of 14.96 and 16.90%, respectively. These results indicated that the addition of ZJW-6 accelerated compost maturation. There was no significant difference between SBC and ABC treatments. However, the carbon and nitrogen content in ABC was lower, likely due to the longer autumn composting period.

The change in ammonium nitrogen levels in spring and autumn compost followed a consistent pattern (Figure 1f). They increased rapidly in the initial stage, which was linked to microbial activity that promoted the decomposition of nitrogen-containing organic matter (Lingying et al., 2023). After reaching a peak, the levels dropped. Three factors contributed to this decline: (1) Conversion to ammonia release, (2) Conversion to nitrogen-containing compounds, such as nucleic acids and proteins, through microbial fixation, (3) Conversion to nitrate nitrogen (Zhang et al., 2016). The ammonium nitrogen in the compost was 3.55%–12.30% lower than that in compost without bacteria. The ammonium nitrogen contents in spring compost were 1.85%–20.53% higher than those in autumn compost. The nitrate nitrogen in spring compost showed an increasing trend, while autumn compost exhibited an increase followed by a decrease (Figure 1g). Nitrate nitrogen contents in spring compost were 2.96–28.03% higher than those in autumn compost. This difference can be attributed to the shorter composting time in spring compared to autumn, which resulted in less loss of ammonium and nitrate nitrogen. In the MP stage, compost with bacteria had 6.18%–17.12% higher nitrate nitrogen levels than compost without added bacteria. This is due to the fact that Firmicutes are higher in added microbial compost than unadded, and Firmicutes play an important role in ammonia-nitrogen conversion and reduction of nitrogen loss (Yin et al., 2019), which results in the conversion of ammonium nitrogen to nitrate nitrogen.

### 3.2 Variation in humic and fulvic acids during composting

**Figure 2:**
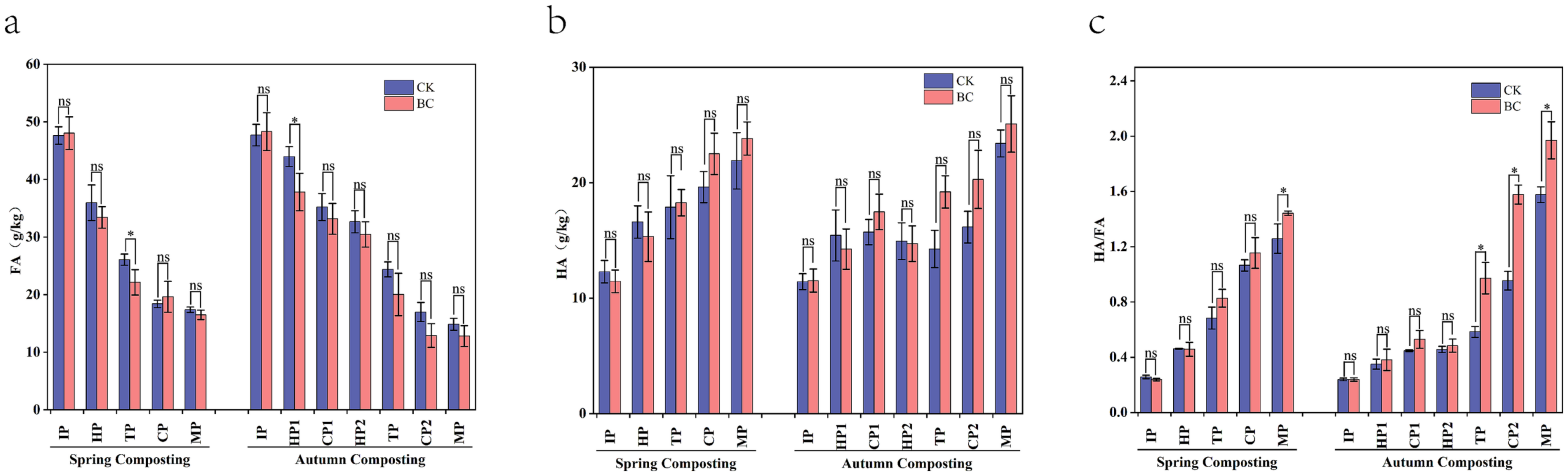
Variation of humification parameters in spring and autumn compost. **(a)** fulvic acid (FA), **(b)** humic acid (HA), **(c)** HA/FA. ^*^Represent significant differences at *p* < 0.05 level.

During the composting process, straw is converted into phenols, carboxyl compounds, amino acids, and polysaccharides (Wang et al., 2024), These compounds are then synthesized into humic substances (HS) by microorganisms and enzymes or through a Maillard reaction (Di et al., 2022). The main constituents of HS are humic acid (HA) and fulvic acid (FA). However, most of the FA is converted into HA due to the instability of FA, which promotes compost maturation (Escobar et al., 2024). In this study, the FA in both spring and autumn composts decreased sharply during the warming phase and reached a minimum at the completion of composting, after which it tended to stabilize (Figure 2a). Specifically, the FA content in SCK, ACK, SBC, and ABC decreased by 63.53%, 68.86%, 65.64%, and 73.50%, respectively. In addition, throughout the composting period, the HA content in all treatments showed an overall increasing trend. The HA content in SCK, ACK, SBC, and ABC increased by 78.11%, 104.71%, 108.00%, and 117.75%, respectively (Figure 2b), spring and autumn composts with bacteria were 8.81 and 7.22% higher in HA than those without bacteria, respectively, which was consistent with previous results (Zhao et al., 2017). Notably, the HA content in SBC was higher than in ACK. This suggests that the addition of ZJW-6 not only promoted the conversion of FA to HA but also shortened the compost decay time. Compared to spring composting, the HA content in autumn composting increased sharply in the late stages. This could be related to the longer duration of autumn composting. After experiencing the entire winter dormancy, microorganisms became active and multiplied, consuming a large amount of HA precursor material. This limited the accumulation of humic acid during the pre-composting phase. The HA/FA ratio is an indicator of compost maturity (Zhang et al., 2018). No significantly, difference (*p* > 0.05) was observed between the individual composts during the IP stage (Figure 2c). During the MP stage, the HA/FA values for SCK, ACK, SBC, and ABC were 1.26, 1.58, 1.44, and 1.97, respectively, all of which met the maturity standard (HA/FA > 1) (Ning et al., 2021b), spring and autumn compost with added bacteria were significantly higher in values (*p* < 0.05) compared to those without bacterial addition, showing increases of 14.29% and 24.68%, respectively. Among them, ABC exhibited the highest HA/FA value, indicating the highest level of humification, the most complete compost treatments, and the most thorough humification process. Meanwhile, inoculation of compound microbial inoculum can shorten the composting time (Peng et al., 2024).

### 3.3 Variation in lignocellulose content and corresponding enzyme activities during composting

**Figure 3:**
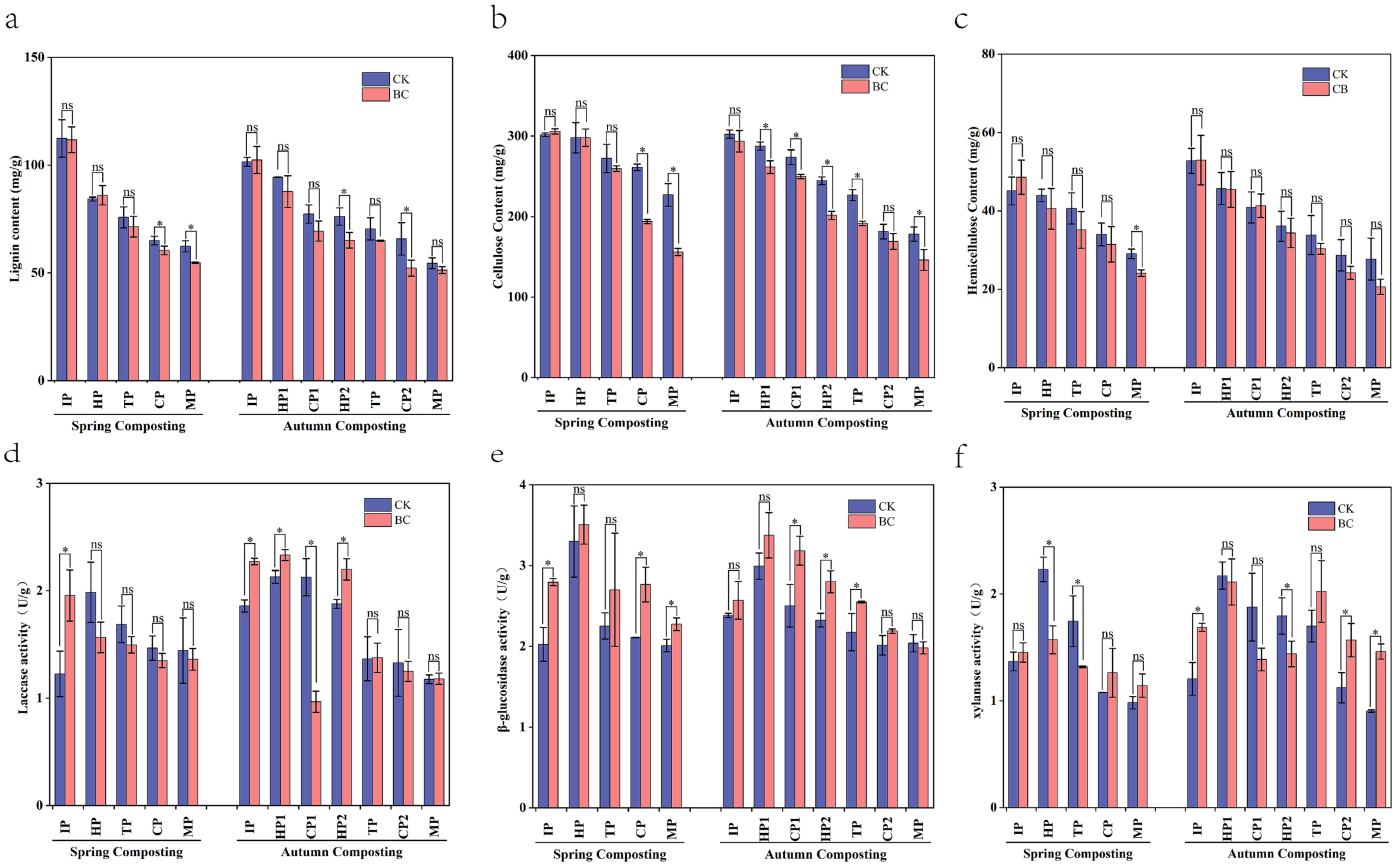
Variation of lignocellulose cellulose content and enzyme activity in spring and autumn compost. **(a)** Lignin content, **(b)** Cellulose content, **(c)** Hemicellulose content, **(d)** Laccase activity, **(e)** *β*-glucosidase activity, **(f)** Xylanase activity. ^*^Represent significant differences at *p* < 0.05 level.

Lignocellulose is a complex polymeric organic matter that resists material degradation (Zhao et al., 2017), This makes the extent of its degradation a key factor in determining compost maturity. In this experiment, lignocellulose content decreased as the composting process progressed (Figures 3a–c), with a notable reduction in the SBC and ABC treatments. At the MP stage of composting, the degradation of lignin, cellulose, and hemicellulose increased by 7.63–14.71%, 22.45–97.76%, and 28.48–41.71%, respectively, when composting with bacteria compared to composting without bacteria. This indicates that the addition of ZJW-6 can enhance lignocellulose degradation efficiency, especially for cellulose and hemicellulose. This improvement is attributed to the differing degradation abilities of the inoculated microorganisms and the influence of the external environment (Di et al., 2024). Moreover, the SBC treatment improved lignin, cellulose, and hemicellulose degradation by 10.22, 19.41, and 6.00%, respectively, compared to ACK treatment, and cellulose was especially significant (*p* < 0.05). This suggests that adding ZJW-6 can effectively shorten composting time and enhance degradation efficiency. It offers potential solutions for agricultural challenges, such as cold weather in northeastern China and the practice of returning straw to fields during spring and autumn. In addition, the cellulose degradation efficiency in the later stage of SBC was significantly improved. This improvement was attributed to the microorganisms added in this experiment. These microorganisms had an optimal cellulose degradation temperature of around 30°C (Lei et al., 2023). On the other hand, the temperature in the late stage of spring composting ranged from 30°C to 40°C. This temperature range was more conducive to the activity of ZJW-6.

Laccase, xylanase, and *β*-glucosidase are key enzymes in the decomposition of lignin, hemicellulose, and cellulose (Shunyao et al., 2022). The changes in the activities of these three enzymes were significantly different after the addition of ZJW-6 (Figures 3d–f). Compared to SCK and ACK, the laccase activity in SBC and ABC peaked earlier. This early peak is highly beneficial for straw degradation. This is because lignin is the main limiting factor in straw degradation. It is embedded between cellulose and hemicellulose, forming a complex and stable spatial structure that inhibits the activity of cellulase and hemicellulase (Wu et al., 2021). Massilia and Paenibacillus were the main advantageous bacteria in the pre-composting period and were able to produce lignocellulolytic enzymes (Nicholas et al., 2016; Raths et al., 2020), which may be the main reason for the increase of laccase. Therefore, the addition of cellulose-degrading bacteria promotes early lignin degradation, creating favorable conditions for further straw degradation. The changes in xylanase activity were opposite to those of laccase. Higher enzyme activities were observed in the later stages of composting in SBC and ABC. Compost with added bacteria showed higher β-glucosidase activity compared to compost without bacterial addition. This increase can be attributed to the accelerated cellulose degradation by the microorganisms. This process led to an increase in fibrous disaccharide products. These products provided sufficient substrates for the enzymatic reaction and enhanced enzyme activity.

### 3.4 Bacterial community succession during composting

**Figure 4:**
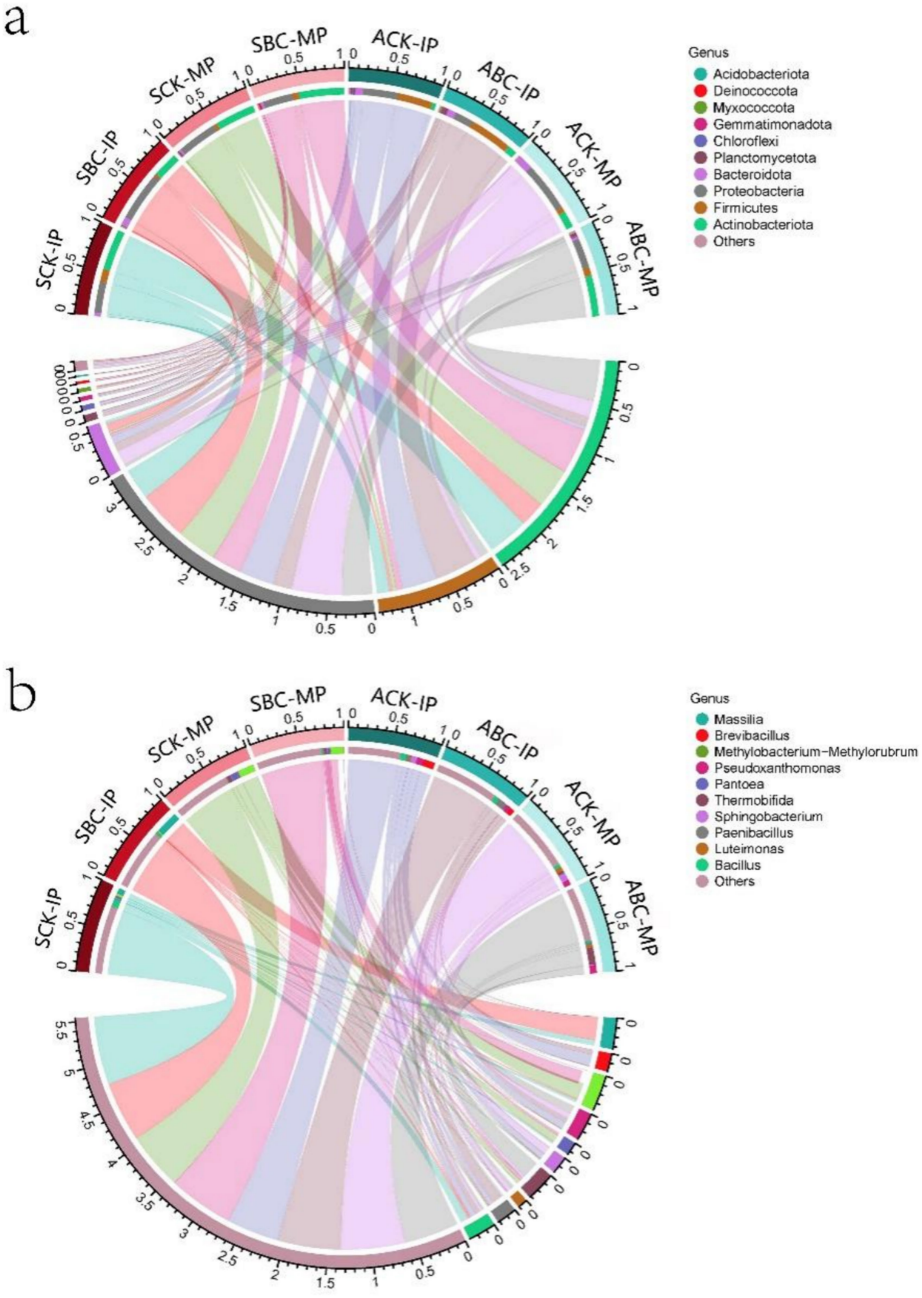
Microbial variation at the level of spring and autumn composting phylum **(a)** and genus **(b)** in the IP and MP phases (Numerical values represent the relative microbial abundances).

The variations in microbial communities across different composts during two periods (IP and MP) were examined using 16S RNA sequencing. Changes in the bacterial community after the addition of microbial agents were assessed by comparing the levels of 10 bacterial genera and phyla before the compost treatments. As shown in Figure 4a, Actinobacteriota, Proteobacteria, and Firmicutes were the dominant bacterial phyla at different stages of spring and autumn composting. The addition of cellulose-degrading bacteria resulted in alterations in bacterial community composition. In the MP stage of composting, Actinobacteriota was the most dominant phylum. It showed a significant increase of 80.14 and 336.08% after the addition of microorganisms in both spring and autumn, with a particularly notable rise in the autumn treatment. This suggested that the addition of cellulose-degrading bacteria reduced nutrient competition from other phyla. This may be due to the addition of ZJW-6 microorganisms elevated the compost temperature, this creates a favorable environment for heat-tolerant Actinobacteriota, which promotes the enrichment of Actinobacteriota (Zhao et al., 2017). Actinobacteriota can secrete lignocellulose-degrading enzymes and various antibiotics (Gen et al., 2021), which promote compost degradation, kill pathogenic bacteria, and play a crucial role in compost quality (Yu-Xin et al., 2021). In addition, they promote carbon retention and increase humic acid content (Ma and Li, 2024). The results indicated that the addition of ZJW-6 enhanced the growth of Actinobacteriota and accelerated compost humification. At the MP stage, Firmicutes in compost with ZJW-6 added were 52.81%–57.91% higher than those without ZJW-6, which could promote ammonia-nitrogen conversion and reduce nitrogen loss (Yin et al., 2019), thus lowering C/N.

During the IP stage of composting, the bacterial communities in SCK and SBC were dominated by Massilia, Bacillus, and Sphingomonas (Figure 4b). However, the bacterial communities in ACK and ABC were dominated by Brevibacillus, Pseudoxanthomonas, Paenibacillus, and Bacillus, with Sphingobacterium also being dominant. After the addition of cellulose-degrading bacteria, the dominant flora of the microbial community in the spring and autumn composting processes changed. In spring composting, the dominant bacteria change from Bacillus to Massilia, while in autumn composting of straw, the dominant bacteria changed from Brevibacillus to Paenibacillus. Notably, genera such as Massilia, Bacillus and Paenibacillus, among others, play a significant role in organic matter degradation (Moura et al., 2018; Yin et al., 2019; Cai et al., 2024). This suggests that the addition of ZJW-6 enhances the microbial community that degrades organic matter and reduce the total carbon of compost. The increase in the relative abundance of Massilia in the spring compost pile was particularly significant, promoting the dominance of different genera in various microbial communities. It is also worth noting that Paenibacillus has the ability to kill nematodes under outdoor conditions (Shi et al., 2024). Meanwhile, Massilia can degrade toxic gases (Son et al., 2021), both of which significantly enhance the overall quality of the compost. There was no difference in microbial community composition during the MP stage of spring straw composting. However, the relative abundance of Methylorubrum was higher in SCK compared to SBC. This difference is likely due to the incomplete humification of the spring compost during degradation. As a result, intermediates such as monocarbons were generated, serving as the primary nutrient source for Methylorubrum (Chauhan et al., 2015). In the MP stage of autumn straw composting, the relative abundance of Thermobifida was higher in ABC compared to ACK. Thermobifida is capable of degrading cellulose and lignocellulosic residues using cellulases (June et al., 2023), and promote the activity of lignocellulases such as laccase and *β*-glucosidase for better degradation of lignocellulose in compost (Jiayu and Xiufen, 2022). The results indicate that ZJW-6 improves the bacterial flora composition, forming a dominant community that facilitates lignocellulose degradation. At the same time, it enhances the compost’s sterilization ability, contributing to the production of higher-quality material for humus formation.

### 3.5 Relationship between environmental factors and microbial communities

**Figure 5:**
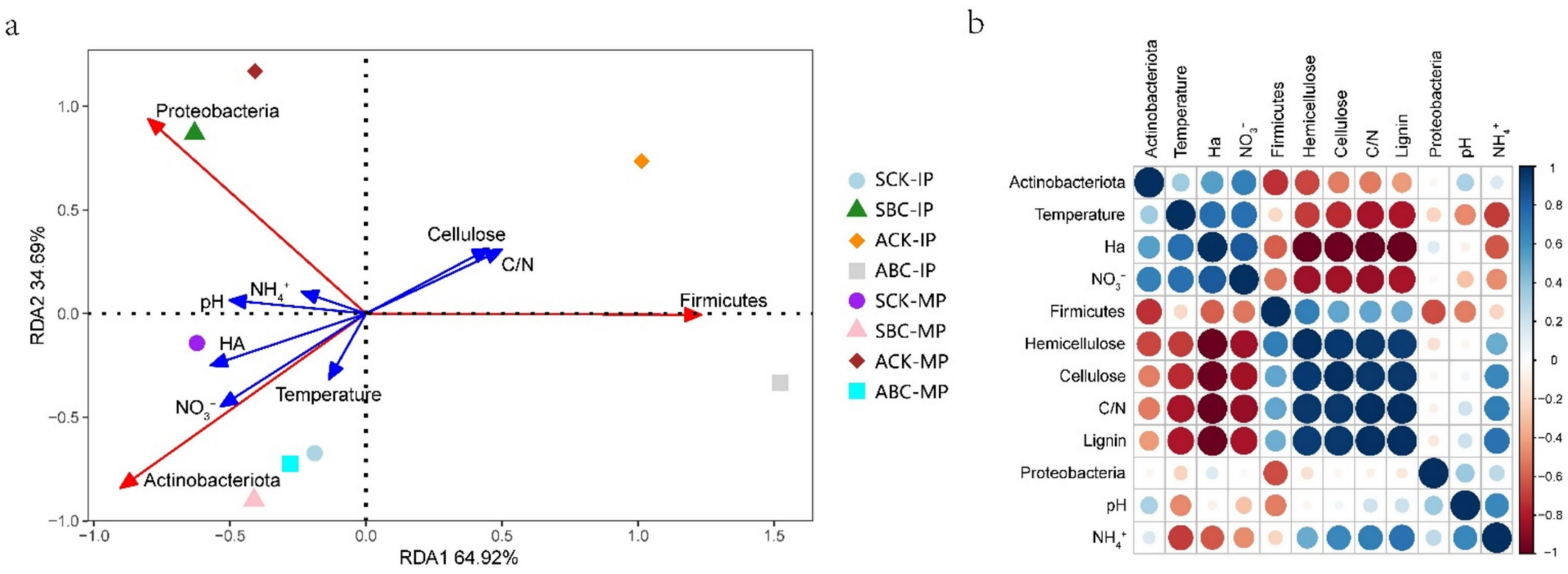
Redundancy analysis (RDA) **(a)**, correlation analysis of environmental indicators and bacterial communities in spring and autumn composting **(b)**.

The composition of the bacterial community is impacted by environmental parameters (Wang et al., 2015). Redundancy analysis (RDA) was used to explain the correlation between different compost treatments, bacterial communities, and environmental indicators (Figure 5a). RDA1 and RDA2 accounted for 34.69% and 64.92% of the variation in bacterial flora in compost, respectively. Actinobacteriota exhibited a negative correlation with lignocellulose. This indicates that, among the three most abundant phyla, Actinobacteriota were the most dominant in decomposing lignocellulose. Furthermore, NO3− had the greatest impact on Actinobacteriota, followed by temperature and humic acid (HA). This suggests that Actinobacteriota is the main phylum contributing to compost warming and putrefaction (Jiang et al., 2019). The addition of ZJW-6 enhanced the correlation between actinomycetes and the spring and autumn compost during the MP stage. This indicates that ZJW-6 increases the abundance of the Actinobacteriota phylum. The microbial phyla at the IP stage of spring and autumn composting were significantly different. However, with the addition of ZJW-6, similar phyla structures were observed at the MP stage of both spring and autumn composting. This indicates that ZJW-6 improves microbial phyla composition and promotes the growth and activity of Actinobacteriota. Figure 5b further confirms that Actinobacteriota showed a negative correlation with lignocellulose and a positive correlation with HA. At the same time, Actinobacteriota promoted NO3^−^ accumulation, NH4^+^ emission, and a reduction in the C/N ratio. Taken together, these findings demonstrate that Actinobacteriota play a critical role in compost maturation throughout the composting process.

### 3.6 Bacterial community co-occurrence network analysis

**Figure 6:**
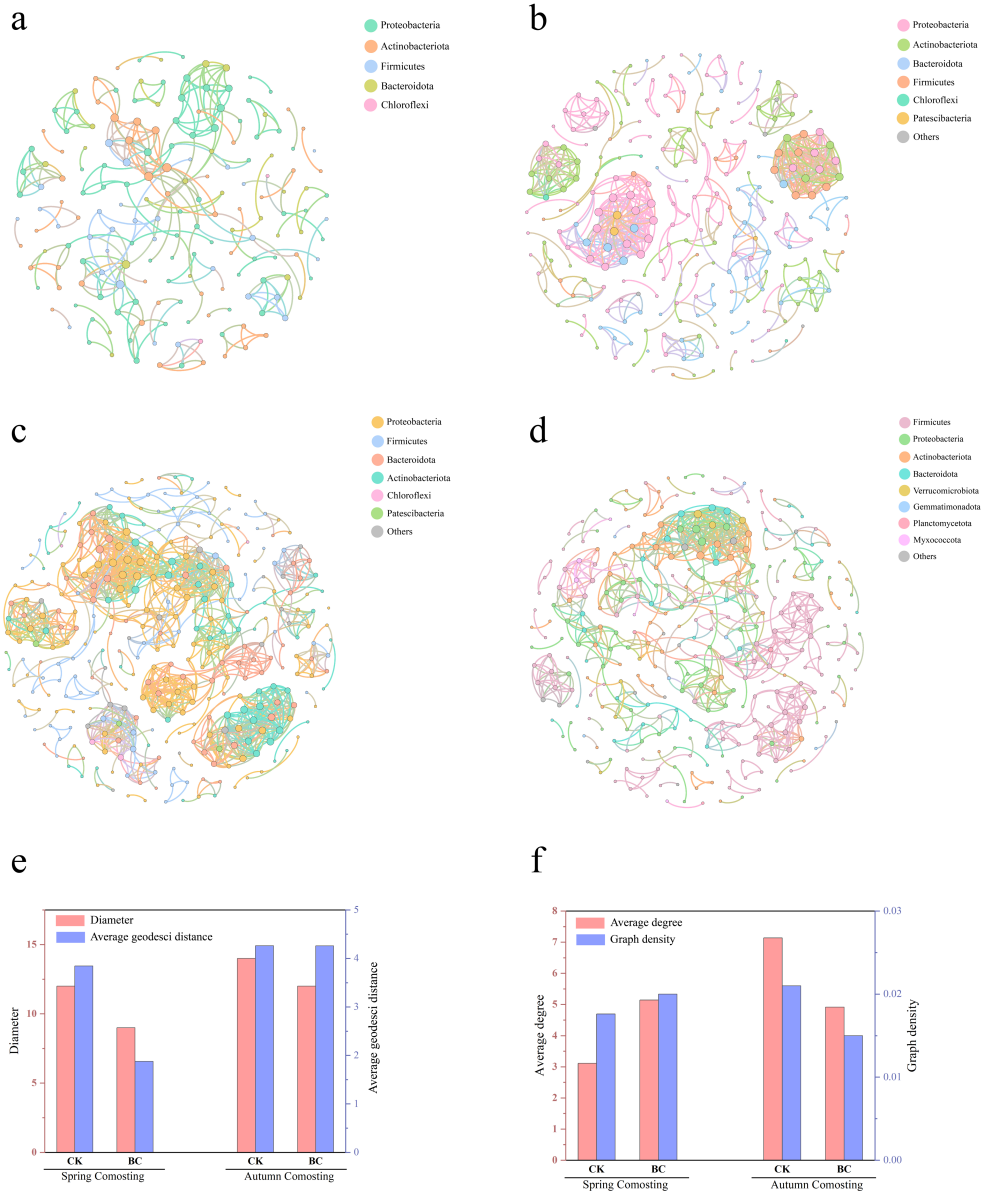
Network analysis of bacterial phylum interactions in SCK, SBC, ACK, and ABC **(a–d)**, Diameter and average geodesic distance of different networks **(e)**, average degree, and graph density of different networks **(f)**.

All OTU data from spring and autumn composting treatments were used to construct co-occurring networks (R > 0.7; *p* ≤ 0.001) to further investigate the mechanisms of bacterial interactions (Figures 6a–d). The networks for SCK, SBC, ACK, and ABC contained 178, 264, 340, and 324 nodes, respectively, with 277, 679, 1,213, and 796 edges. The diameters and average geodetic distances of the SCK and ACK treatments were higher than those of the SBC and ACK treatments. This suggests that these parameters reflect the intensity of interactions between microorganisms (Xinguang et al., 2022). This indicates that microorganisms in the SCK and ACK treatments continued to contribute to compost degradation, likely due to incomplete fermentation. The average degree and density of the spring and autumn composts showed opposite trends after the addition of microorganisms (Figures 6e–f). This may be attributed to the different microbial compositions at the start of the spring and autumn composts. Notably, spring compost exhibited enhanced microbial interrelationships after the addition of ZJW-6. In contrast, autumn compost showed a decline in interrelationships. This was possibly due to excessive degradation in the ABC treatments, which led to the dominance of certain species.

## 4 Conclusion

The addition of the fungi-static *C. iranensis* (ZJW-6) enhanced the microbial composition of the compost by promoting the relative abundance of Actinobacteriota and lignocellulose-degrading bacteria. This fostered a dominant flora that accelerated lignocellulose degradation, reduced the C/N ratio, and increased both humic acid content and nitrogen nutrients, thereby improving compost quality. Longer composting times (autumn composting) further facilitated straw degradation. Therefore, adding ZJW-6 bacteria to autumn composting is the most effective method for straw composting and field application in Northeast China. In addition, the inclusion of ZJW-6 bacteria in spring straw composting achieved a decomposition level comparable to that of autumn straw composting without the addition of bacteria. This provides a technical foundation for spring straw composting. Overall, we recommend autumn composting with ZJW-6 as the preferred strategy for straw return in Northeast China. However, in practical field applications, ZJW-6 is susceptible to environmental influences and is not as controllable as composting inoculum, leading to a decrease in degradation efficiency.

## Data Availability

The raw data supporting the conclusions of this article will be made available by the authors, without undue reservation.
